# Decellularized Liver Matrices for Expanding the Donor Pool—An Evaluation of Existing Protocols and Future Trends

**DOI:** 10.3390/biom15010098

**Published:** 2025-01-10

**Authors:** Marcin Morawski, Maciej Krasnodębski, Jakub Rochoń, Hubert Kubiszewski, Michał Marzęcki, Dominik Topyła, Kacper Murat, Mikołaj Staszewski, Jacek Szczytko, Marek Maleszewski, Michał Grąt

**Affiliations:** 1 Department of General, Transplant, and Liver Surgery, Medical University of Warsaw, 02-091 Warsaw, Poland; mwkrasn@gmail.com (M.K.); kuba.rochon@gmail.com (J.R.); hubertkubiszewski23@gmail.com (H.K.); mst.staszewski@gmail.com (M.S.); michal.grat@gmail.com (M.G.); 2 Institute of Telecommunications, Warsaw University of Technology, 00-665 Warsaw, Poland; michal.marzecki@pw.edu.pl (M.M.); dominiktopyla@gmail.com (D.T.); kacpermurat@gmail.com (K.M.); 3 Institute of Experimental Physics, Faculty of Physics, University of Warsaw, 02-093 Warsaw, Poland; jacek.szczytko@fuw.edu.pl; 4 Department of Embryology, Institute of Developmental Biology and Biomedical Sciences, Faculty of Biology, University of Warsaw, 02-096 Warsaw, Poland; ma.maleszewski@uw.edu.pl

**Keywords:** bioengineered organs, decellularized liver, liver transplantation, tissue engineering, regenerative medicine

## Abstract

Liver transplantation is the only curative option for end-stage liver disease and is necessary for an increasing number of patients with advanced primary or secondary liver cancer. Many patient groups can benefit from this treatment, however the shortage of liver grafts remains an unsolved problem. Liver bioengineering offers a promising method for expanding the donor pool through the production of acellular scaffolds that can be seeded with recipient cells. Decellularization protocols involve the removal of cells using various chemical, physical, and enzymatic steps to create a collagenous network that provides support for introduced cells and future vascular and biliary beds. However, the removal of the cells causes varying degrees of matrix damage, that can affect cell seeding and future organ performance. The main objective of this review is to present the existing techniques of producing decellularized livers, with an emphasis on the assessment and definition of acellularity. Decellularization agents are discussed, and the standard process of acellular matrix production is evaluated. We also introduce the concept of the stepwise assessment of the matrix during decellularization through decellularization cycles. This method may lead to shorter detergent exposure times and less scaffold damage. The introduction of apoptosis induction in the field of organ engineering may provide a valuable alternative to existing long perfusion protocols, which lead to significant matrix damage. A thorough understanding of the decellularization process and the action of the various factors influencing the final composition of the scaffold is essential to produce a biocompatible matrix, which can be the basis for further studies regarding recellularization and retransplantation.

## 1. Introduction

Chronic liver disease affects around 29 million people in Europe, and its most severe form—cirrhosis—is responsible for 170,000 deaths each year [1]. The only curative treatment for end-stage liver failure is orthotopic liver transplantation (LTx), with more than 5500 of these procedures performed each year in Europe [1]. Although new data continue to identify new patient groups that could benefit from LTx, the number of potential liver donors has recently plateaued. Thus, this increased donor–recipient discrepancy may account for up to 20% of all cases of waiting list mortality [2].

Decellularized organ matrices constitute a promising solution to donor organ shortage and waiting list mortality. This approach was first described in the early 1990s by Badylak et al., who successfully produced porcine small intestine submucosa (SIS) devoid of cellular material [3,4]. The first clinical application of SIS was in rotator cuff repair, followed by numerous studies using SIS in the abdominal wall, the heart valve, and the bladder, as well as in penile repair [5,6,7]. Promising prospects for the clinical application of decellularized SIS led to the development of decellularization protocols for whole organ specimens. In 2008, Ott et al. reported the production of an acellular rat heart that retained its macro- and microscopic architecture. After perfusion with neonatal rat cardiomyocytes and endothelial cells, the recellularized rat heart was able to generate a contractile force of 2.4 mm Hg (2% of adult rat heart function) [8]. The very first reports on liver decellularization were published simultaneously in 2010 by Uygun and Shupe [9,10]. The liver matrices obtained were shown to maintain their internal microscopic structure, vasculature, and biocompatibility. These and subsequent studies have shown that the acellular liver matrix is able to drive different cell lines towards the creation of hepatocyte-like cells [9,10,11]. Because cells from the recipient can be reintroduced and the matrix components possess low immunogenicity, transplanted biofabricated organs should not require immunosuppressive protocols, the side effects of which can contribute to patient morbidity and mortality following LTx.

Elements of the decellularized matrix provide an environment for cell movement, adhesion, and differentiation, and the maintenance of the shape and turgor, and also provide the mechanical strength of the scaffold [12]. Collagen I is the most abundant component of the matrix, accounting for up to 90% of its dry weight [13]. Other types of collagen, mainly collagen IV and VII, provide support for the vessel walls to maintain their shape and patency. The spaces between collagen fibers are filled with numerous compounds that interact with other matrix components and cells. Glycosaminoglycans (GAGs) are capable of binding many cytokines and growth factors. Thanks to their numerous sulphate moieties, GAGs promote water retention, maintain organ turgor, and prevent matrix contraction [14]. Because cells cannot interact directly with the matrix collagen and GAGs, several proteins are required for cell–matrix contact. Fibronectin and laminin beta-2 are examples of such proteins, which mediate the interaction of stromal and vascular cells with scaffold structures [15,16].

In the production of acellular organs, the aim should be to obtain a collagenous matrix free of cell debris while minimizing exposure to decellularization agents that damage residual proteins and wash out vital matrix components. Further optimization of this process requires a thorough understanding of the mechanisms of action of decellularization agents. Existing data from animal models confirm the feasibility of producing biocompatible liver matrices using various protocols but often do not allow for systematic comparison. In this review, we described and analyzed the most common decellularization agents and protocols. Based on the existing protocols, we established the major drawbacks and limitations of the existing methods used to produce and assess acellular matrices. We also identified methods that may allow for the production of decellularized matrices with little disruption to their native components.

## 2. Decellularization Overview

The concept of decellularization is based on the removal of cellular material from native tissue, leaving the extracellular matrix (ECM) intact. The ECM provides a 3D scaffold for further cell seeding and is rich in adhesion sites and growth factors that support the phenotype and survival of cells. Unlike synthetic materials, decellularized matrices retain large vessels that can aid the surgical technique of organ implantation. However, the process of cell removal can damage the remaining ECM, washing out important components of the matrix and destroying the fragile ECM microarchitecture. Therefore, the subsequent process of recellularization does not lead to the ideal reconstruction of the native organ.

### 2.1. Definition of Acellular Matrix

There is currently no quantitative definition of a decellularized liver matrix. The mere macroscopic appearance of a translucent, whiteish organ does not seem to be sufficient as the matrix may still contain the DNA and cellular debris of the donor [17,18]. The presence of these may cause adverse reactions in recipients and may be unacceptable when considering the use of animal organ matrices [19,20]. Remaining DNA fragments can activate immunological system and elicit an inflammatory response directed against the newly engineered organ [21,22]. On the other hand, prolonged exposure to decellularization agents can cause excessive matrix damage and the leaching of structural components and growth factors. The decellularization criteria proposed by Crapo et al. aimed to provide a quantitative definition of acellular matrix, but this is not widely accepted [23]:

• A lack of visible nuclear material (H&E or 4′,6′-diamidino-2-phenyloindole [DAPI] staining);
• DNA fragments no longer than 200 base pairs;
• A dsDNA concentration of less than 50 ng per mg ECM dry weight.

**Figure 1:**
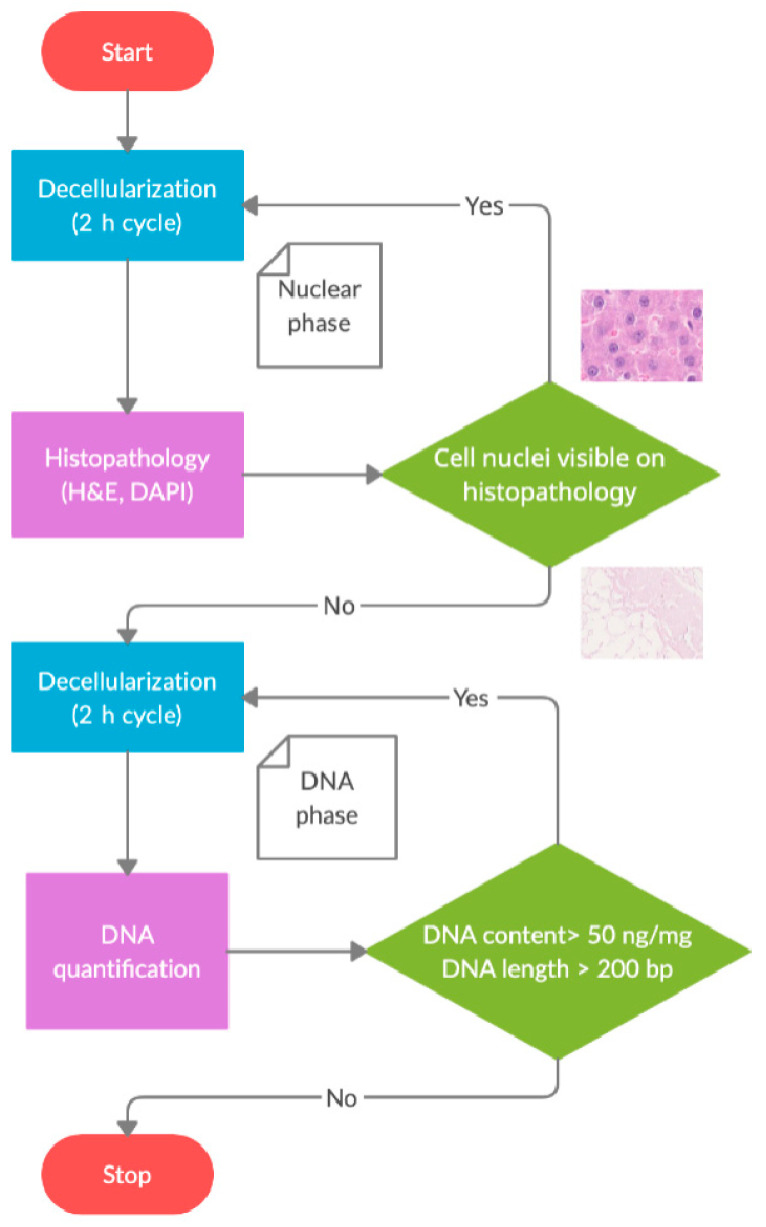
An overview of the decellularization cycles. [Author’s own work].

Based on basic histological techniques, colourimetric assays, and gel electrophoresis, these criteria provide a rapid and reproducible method of matrix assessment. However, there is no protocol that allows for the comparison and optimization of the decellularization process. As prolonged exposure to decellularization agents leads to the washout of matrix components, it seems important to be able to determine the optimal time for organ perfusion. To solve this problem, we proposed the protocol of decellularization cycles, which would help when comparing the effectiveness of different processes (Figure 1).

**Figure 2:**
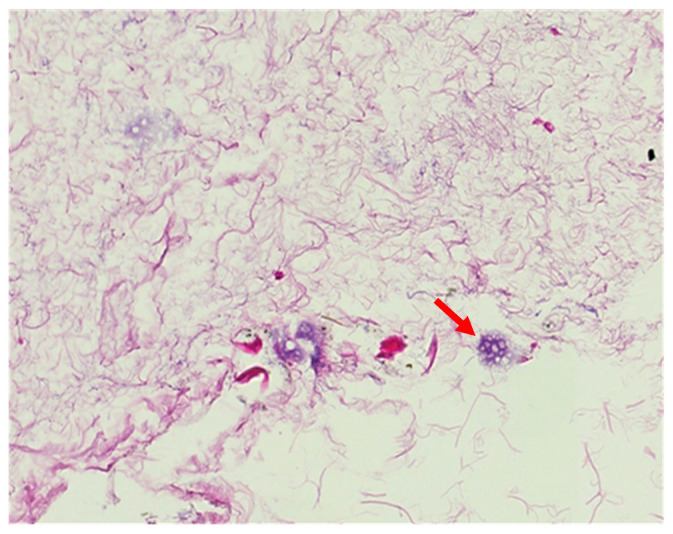
A fragment of a putative acellular liver matrix after 24 h of perfusion with a 0.1% sodium dodecyl sulphate (SDS) solution. The red arrow points at the cell nucleus entrapped in the matrix fibers. [Author’s own work].

To determine the optimal timeframe for the decellularization protocol, the process should be divided into two phases: (1) a nucleic phase and (2) a DNA phase. Each phase consists of 2 h decellularization cycles. In both the nucleic and DNA phases, each cycle is followed by an intermission. During the intermission, a core needle biopsy is taken from the organ. First, in the nucleic phase, the biopsy should be evaluated for the presence of cell nuclei on frozen section specimens using H&E or DAPI stainings. Nucleic phase decellularization cycles should be repeated until no nuclei and DNA fragments are visible in either staining. This observation justifies transition into the DNA phase. Similarly, the quantitative assessment of DNA and DNA fragment length should follow each cycle in the DNA phase. Decellularization cycles should be repeated until the criteria for DNA content are met, namely (1) <50 pg per 1 mg dry weight ECM; and (2) no DNA fragments greater than 200 bp in length. Figure 2 shows an example of the presence of cell nuclei in a putative acellular liver matrix, highlighting the need for a two-step assessment of decellularized matrices.

**Figure 3:**
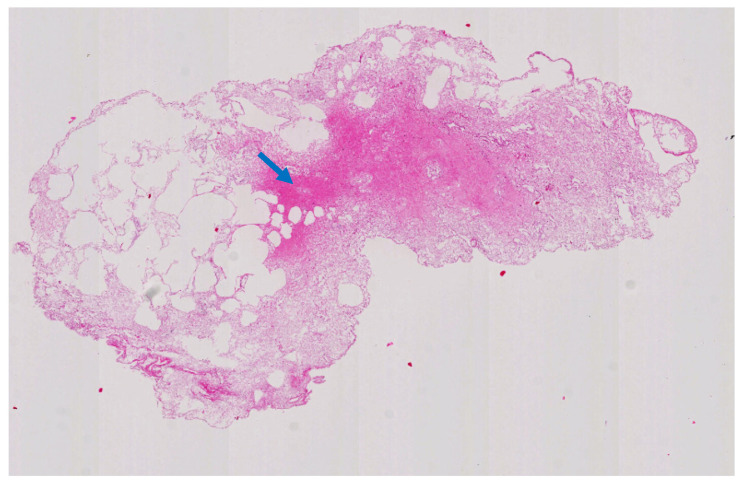
The accumulation of cellular debris in the outlet area of a decellularized rat liver (blue arrow). [Author’s own work].

The standardized protocol for liver decellularization should include the sampling of different areas of the organ. In particular, the thin margins and segments adjacent to the hepatic veins should be assessed during the periods between the phases of decellularization. The former area is characterized by poor vascularity and often remains brownish, while the remaining parts of the organ are translucent or whiteish, indicating macroscopically cellular and acellular regions, respectively. The latter area constitutes an outlet for the abundant cellular material that is washed away and may accumulate vast amounts of DNA and proteins (Figure 3).

Moreover, the concept of decellularization cycles is universally applicable, provided that the analyzed parameters indicating efficiency are quantitative. As Hussein et al. point out, although DNA content is commonly used as a surrogate marker for cell removal, it is not a reliable indicator of complete decellularization, and available products containing acellular tissues are not required by the Food and Drug Administration to report on their DNA measurements [24]. The definition of decellularized tissue should also include the potential toxicity of the decellularization agents that may interfere with cell seeding. The presence of other factors, such as alpha-Gal epitopes or porcine endogenous retrovirus, should also be considered when using porcine xenografts [25,26]. On the other hand, a sample of liver tissue should be taken from the organ during the process of decellularization using invasive methods in order to determine its cellularity. This constitutes an obstacle when considering repeated sampling of the organ and can lead to the uncontrolled leakage of perfusion fluid. The non-destructive monitoring of organ decellularization provides an alternative to matrix-destroying intermissions. Such an approach was proposed by Geerts et al., who used computed tomography and efflux perfusate analysis to determine the decellularization status of organs [27]. The non-destructive monitoring of the process may also allow for the real-time assessment of the purity of the matrix and the individualization of the decision to finish the decellularization process.

### 2.2. Perfusion Route

Three main vessels can be used for whole liver perfusion: the portal vein (PV), the hepatic artery (HA), or the inferior vena cava (IVC). The PV has been the most commonly chosen perfusion route, and there are a few reports of simultaneous PV and HA perfusion [28,29]. Struecker et al. showed superior results of HA perfusion compared to PV perfusion in terms of cell removal from the matrix [30]. In addition, livers perfused via the PV under oscillating pressure conditions showed a reduced number of residual cell clusters compared to standard perfusion [28]. Therefore, it can be hypothesized that perfusion via the HA alone is associated with different perfusion dynamics and higher perfusion pressure, as compared to perfusion via the PV. There are single reports on whole organ perfusion through the IVC or the bile duct [10,31]. Simultaneous perfusion via the PV and the HA appears to be the most appropriate route due to its effectiveness in removing cellular material and the ability to monitor perfusion pressure in both vessels, whereas the ICV provides a wide outlet for cellular debris. Monitoring perfusion dynamics may provide a better understanding of the process given the potential changes in the matrix resistance (as shown in Figure 3) and should therefore be routinely included in research protocols. The bile duct does not appear to be suitable for whole liver decellularization due to the lack of anatomical connections between the biliary tree and the liver sinusoids and the associated potential damage caused by pressure build-up in blindly terminated ducts.

### 2.3. Decellularization Protocol

A typical protocol includes several perfusion steps with different decellularization agents. Most commonly, after storage at −80 °C and thawing, livers are perfused with Phosphate Buffered Saline (PBS) or distilled water in order to remove residual blood and apply a hypotonic solution to facilitate cell breakdown. This is followed by the application of active decellularization agents that interact with the cell membrane, dissolving it and releasing the cellular content. To facilitate cell detachment, chelating agents such as ethylenediaminetetraacetic acid (EDTA) are added in order to remove ions involved in cell-matrix interactions [32,33,34,35]. Sodium dodecyl sulfate (SDS) and Triton X-100 are the most commonly used surfactants; however, numerous studies have reported on different active agents including acids, bases, and alcohols, as well as physical stimuli [36,37,38]. The active agent can be applied once or in cycles interrupted by wash steps (usually PBS or dH2O) and at increasing or decreasing concentrations [31,39,40]. As has been shown by Pan et al., the use of gradient concentrations of ionic detergents (SDS 1%, 0.5%, and 0.1%) can result in a shorter perfusion time [41]. In addition to active decellularization agents, various excipients and drugs such as acids, bases, enzymes, or antimicrobials can be added to the perfusion solution. The acellular matrix may then be modified in order to increase its mechanical properties and hemocompatibility (e.g., heparin) as well as to modify immunogenicity through the use of crosslinking agents (glutaraldehyde, genipin) or nanoparticles [42,43,44,45].

### 2.4. Models for Matrix Assessment

A promising rodent model of in vivo decellularization has been demonstrated by perfusing a single liver lobe with a detergent solution, followed by reperfusion [46]. The model demonstrates that the internal matrix vasculature can temporarily maintain physiological blood flow and provides a valuable tool for further studies on matrix biocompatibility and organ recellularization. Given the advantageous gross anatomy of the porcine liver and the segmentation of its parenchyma, the rodent model needs to be validated in this large animal model. Such a model would allow for the assessment of matrix immunogenicity and remodeling in vivo, and appears to be superior to the existing models of subcutaneous matrix implantation [47,48,49]. Similarly, in contrast to complex bioreactors or static 3D culture systems, the porcine model would provide a reliable and simple in vivo alternative [9,50,51,52,53,54].

## 3. Decellularization Agents

Full-size organs require a complex decellularization protocol that allows for the complete washout of cellular material while maintaining the ECM composition and micro-structure. It usually involves the sequential application of decellularization agents that produce the acellular material with minimal undesired effects. Liver decellularization is achieved through organ perfusion with detergent solutions for several hours using peristaltic pumps. The protocols and applied decellularization agents are summarized in the Supplementary Materials (Table S1).

### 3.1. Physical Agents

#### 3.1.1. Freezing

Organ freezing is mainly used to prevent autolysis but also acts as a mild decellularizing agent due to intra-cellular crystal formation. Organs that are to be decellularized are usually stored at −80 °C and are slowly thawed at 4 °C for several hours before decellularization. Freezing is usually uncontrolled and can result in varying degrees of damage to the ECM. The freezing rate appears to be an important factor to consider when designing an optimal decellularization protocol. Bischof and Rubinsky have shown that snap freezing in liquid nitrogen (~1000 °C/min) leads to the formation of large ice crystals in cell nuclei [55]. This phenomenon could increase cellular damage and facilitate the washout of cellular debris due to pre-processing damage to the DNA strands and the nuclear wall. However, this study did not evaluate the effect of snap freezing on ECM crystal formation. Freeze–thaw cycles can also be used to decellularize tissues, although their effectiveness is most evident in certain types of tissue such as tendons that contain relatively few cells or are composed of a dense collagen network that is immune to repeated damage to the ECM [56,57,58,59]. In the context of vascularized organs with a complex internal structure, the importance of freezing may be limited to organ preservation prior to decellularization [60]. This seems particularly important when considering the decellularization of the liver, as its microarchitecture supports arterial, portal, biliary, and outflow structures. The use of cryoprotectants such as dimethyl sulfoxide or trehalose has been proposed to optimize decellularization using freeze–thaw cycles [61,62,63]. This approach provides better preservation of the matrix’s vascular network and may allow for repeated freeze–thaw cycles [64]. Although a single freeze–thaw cycle does not cause DNA degradation, the application of multiple cycles may lead to significant damage to the strands [65]. This phenomenon can be enhanced by maintaining an alkaline pH during the process that causes DNA denaturation [66].

#### 3.1.2. Flow Rate and Perfusion Pressure

**Figure 4:**
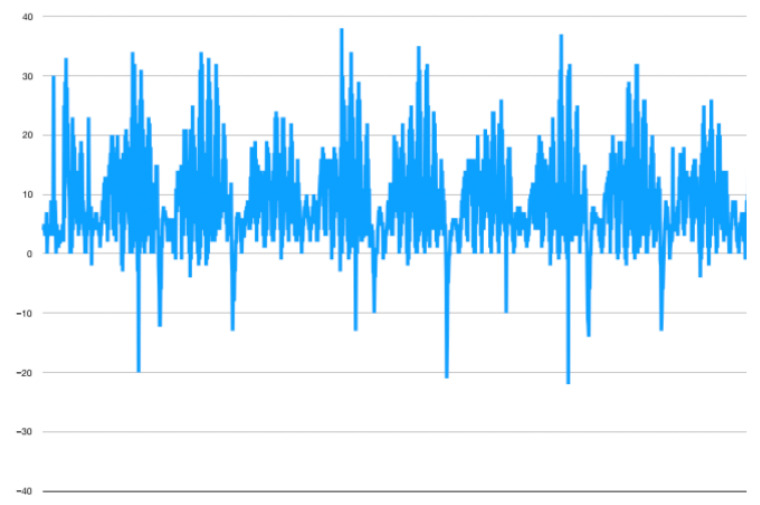
Four full cycles of a standard peristaltic pump with three rollers (3 peaks per cycle; 30 rounds per minute; *y*-axis—pressure [mbar]). Negative values of the flow rate represent the backward flow of the fluid. [Author’s own work].

In the majority of protocols for organ decellularization in rodent, pig, and human models, fluid is circulated through the organ using a peristaltic pump. The main perfusion parameter reported is the flow rate (mL/min), which expresses the average flow through the organ per unit of time. However, the flow rate does not accurately reflect the fluid’s effect on the organ, as the flow is cyclical and oscillatory in nature, a phenomenon illustrated in Figure 4. The characteristics of this flow may affect the efficiency of the decellularization process.

**Figure 5:**
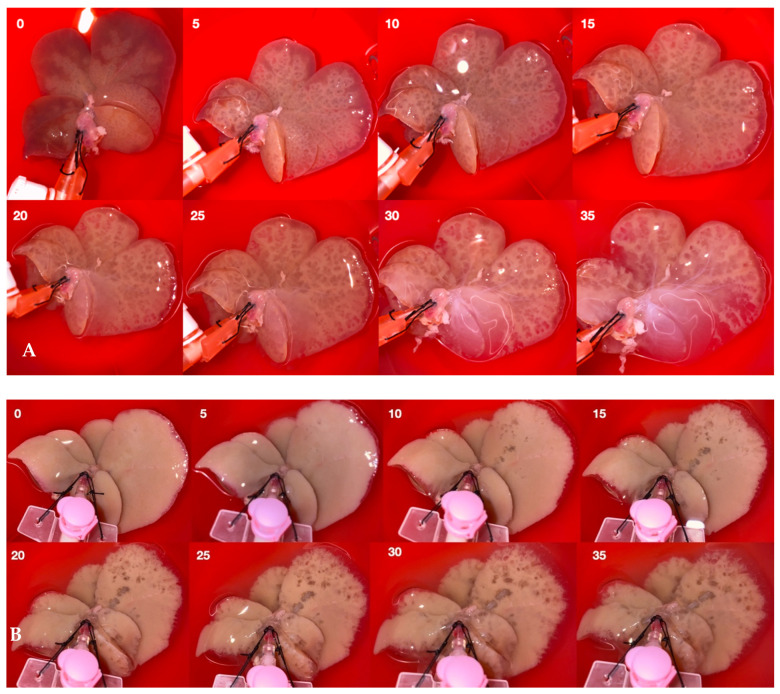
The first 35 min of gravity-driven (pressure of 9 cm H_2_O) (**A**) and peristaltic pump-based (flow rate of 5 mL/min) protocols (both with 0.1% *v/v* SDS) (**B**). [Author’s own work].

In addition, most publications on decellularization in rodent models report a flow rate between 1 and 10 mL/min [9,10,27,28,32,33,34,39,41,67,68,69,70,71]. However, it is difficult to compare these protocols because of the effects of the uncontrolled flow rate and shear stress on the integrity of the collagen network and the composition of the ECM. As shown in the Supplementary Materials (Video S1) and in Figure 4, there is significant backflow during about one third of the pump cycle due to the negative pressure in the deformed silicone tubes. As presented by Hillebrant et al. placing the liver undergoing decellularization in an oscillating pressure chamber that mimics physiological changes during diaphragm contraction and relaxation, enhances the effectiveness of DNA removal [28]. Similar results have been shown by the same team in a porcine model [30]. It appears that the negative pressure created by the pump can be counteracted by the application of external negative pressure, facilitating the onward flow of fluid and thus promoting decellularization. The need for alternating pressure within the decellularization chamber can be easily eliminated thanks to gravity-driven perfusion, as shown by Robertson and Morales-Guerrero [40,72]. Momtahan et al. proposed an automated pressure control system for decellularizing porcine hearts [73]. The authors found that 6 h of detergent exposure for 24 h of perfusion was effective in removing 98% of DNA, but with the application of a peristaltic pump. Most of the porcine and human protocols report pressure-driven perfusion models, but similar to the small animal models, they use peristaltic pumps [16,29,35,45,74,75]. Figure 5 shows a comparison of the first 35 min of gravity-driven (9 cm H_2_O) (Figure 5A) and peristaltic pump-based protocols (Figure 5B) of whole rat liver decellularization. It can be seen that the steady pressure-driven protocol leads to more homogenic and faster macroscopic decellularization. The peristaltic pump-based protocol seems less homogenous and results in slower washout of the cellular material from the organ.

#### 3.1.3. External Pressure

High hydrostatic pressure (HHP) can be used to destroy cells while leaving the extracellular matrix intact. This method involves the application of high pressures of up to 250 MPa and has been shown to be effective in destroying cellular material in cell suspensions [76,77,78]. The cell structures can be denatured and destroyed without damaging the microstructure in the collagen lattice thanks to moderate HHP (100–200 MPa) after a supercooling pretreatment [76]. In addition, thanks to Pascal’s law, the decellularization stimulus is evenly distributed throughout the matrix vasculature, ensuring a uniform effect on the majority of the cells. This method needs to be further validated in the decellularization of parenchymal organs. On the other hand, the application of external negative pressure can also be used to remove the residual detergents from the matrix [79].

### 3.2. Chemical Agents

#### 3.2.1. Hypotonic Solutions

Most decellularization protocols include perfusion steps with hypotonic solutions (e.g., dH_2_O). These osmotically active fluids cause cell swelling and rupture, initiating cell degradation with minimal matrix damage and no toxic residues. However, while these protocols are effective in removing cellular material, they do not completely remove cellular structures from the matrix and cannot be used alone, especially in whole organ models [80]. Secondly, the release of cellular contents without an innate enzyme blockade can damage matrix structural proteins [68]. Hypotonic solutions are also used to remove cellular debris following the use of more potent decellularization agents and to wash out toxic residues such as SDS and Triton X-100. Similarly, the washout of apoptotic bodies may be essential for apoptosis-assisted decellularization (see below, Section 3.3.2) [81,82,83].

#### 3.2.2. Detergents

The amphiphilic structure of detergents is responsible for cell membrane disruption. As the monomers enter the lipid bilayer, they tend to accumulate on the outer layer due to the very slow flip–flop rate. Eventually, they begin to alter the geometry of the membrane thanks to their conical shape, leading to the breakdown of the cell membrane [84]. In the context of decellularization, the monomeric form of detergents appears to play the most important role in solubilizing cell membranes. The appropriate choice of detergent concentration seems to be critical for the effectiveness of decellularization. Increasing the detergent concentration beyond the critical micelle concentration (cmc) does not enhance the efficiency of the process. At higher concentrations, the integration of the monomeric form into the lipid bilayer competes with the self-assembly of the monomers into homogeneous micelles.

Detergents used for decellularization can be divided into ionic, non-ionic, and zwitterionic types, with SDS (anionic) and Triton X-100 (non-ionic) being the most commonly used in the liver decellularization protocols. The cmc for both detergents is estimated at 0.17–0.23% *w/v* for SDS and 0.2% *w*/*w* for Triton X-100 [85,86]. The use of SDS is more efficient in removing cell nuclei than other detergents, but its destructive effect on the ECM is more pronounced [14,23,24]. SDS is also a denaturing agent that interacts with the tertiary structure of proteins. Unlike Triton X-100, SDS can block the activity of the released intra-cellular enzymes, thus protecting the matrix from uncontrolled damage. The importance of blocking the enzymatic activity of enzymes such as matrix metalloproteinases (MMPs) was recently demonstrated by Kasravi et al., who used doxycycline—an antimicrobial and MMP inhibitor—during liver decellularization [68]. However, the interaction of SDS with the structure of collagen fibers may lead to the exposure of cryptic antigens and increase the immunogenicity of the matrix upon implantation [23,24,87]. Triton X-100, used as the sole decellularization agent, was insufficient for producing acellular matrices [88]. Similarly to SDS, Triton X-100 has also been shown to increase matrix reactivity by cleaving DNA into larger fragments than SDS [89,90]. Since even small amounts of SDS (>10 µg/mg dry weight) are toxic to cells, a method to completely remove the detergent is critical. Triton X-100, together with CaCl_2_ can be used to remove residual SDS from the matrix [9,91]. A comparison of SDS and Triton X-100 perfusion in the context of liver decellularization was presented by Ren et al., and both SDS and Triton X-100 appeared to be effective in producing acellular matrices [71]. SDS-treated scaffolds were shown to have lower levels of elastin (20% vs. 60%), GAG (10% vs. 50%), and the hepatocyte growth factor (20% vs. 60%) as compared to Triton X-100-treated organs. In addition, treatment with Triton X-100 was associated with improved scaffold biocompatibility and the support of liver-specific functions such as albumin secretion, urea synthesis, ammonia elimination, and cytochrome p450 mRNA expression after recellularization.

#### 3.2.3. Acids and Bases

Acidic and alkaline solutions can enhance decellularization when used in combination with other active agents, but their effects alone are limited. Peracetic acid is one of the most commonly used substances for both the decellularization and sterilization of matrices [14,23,34]. However, it is known to reduce the mechanical strength of matrices and remove large amounts of GAGs and growth factors [36,92,93]. Bases such as sodium or ammonium hydroxide are often added to the active agents to facilitate DNA washout by denaturing the helix [16,69,71]. However, prolonged exposure to alkaline agents can lead to matrix disruption due to the destruction of collagen fibrils and crosslinks [94].

### 3.3. Biological Agents

#### 3.3.1. Enzymes

Similar to the detachment of adherent cell lines in culture, enzymes such as trypsin can be used to dissociate cells from the ECM [95]. However, due to its non-selective action on peptide bonds at the carboxyl group of lysine and arginine, it can cause significant matrix damage [96]. Ultrastructural analysis of decellularized livers showed significant damage to the ECM in organs treated with protocols containing trypsin [88]. In addition, its activity is inhibited by protease inhibitors released from ruptured cells [24]. Contrary to trypsin, nucleases act predominantly on DNA, breaking large fragments into shorter strands that can move more freely in the collagenous network, as observed in gel electrophoresis. Ahmed et al. showed that the addition of DNase to Triton X-100 improved the performance of the decellularization protocol while maintaining biocompatibility and preserving the matrix ultrastructure [88]. In a large animal model, Bühler et al. also confirmed that livers treated with SDS + DNase contain less DNA than those treated with SDS alone (135 ± 44 ng/mg dry weight ECM vs. 359 ± 89 ng/mg dry weight ECM) [75]. They also showed a higher GAG concentration in organs decellularized with the former protocol (5.3 ± 1.17 μg/mg dry weight ECM vs. 3.22 ± 0.71 μg/mg dry weight ECM).

#### 3.3.2. Apoptosis

Apoptosis is a form of programmed cell death that disposes of the components of cells in small vesicles without the inflammation and release of cell content. Controlled cell destruction through induced apoptosis before organ harvesting could potentially reduce the detergent exposure time required later for successful decellularization. The so-called death receptor Fas (CD95/APO-1) is responsible for the elimination of senescent, infected, or neoplastic cells from the liver. The stimulation of hepatocyte apoptosis through the Fas-induced pathway in an animal model has been described by Faletti et al. [97] Hepatocyte sensitization to an apoptotic stimulus can be achieved through pretreatment with TNF-α. The subsequent administration of the Fas ligand or the anti-Fas agonistic antibody was shown to promote massive hepatocyte apoptosis. This method, although limited to animal models, may allow extensive cell elimination prior to perfusion steps, thereby reducing the time required to decellularize the organ and potentially preserving the native composition of the ECM.

## 4. Discussion

The production of acellular matrices should result in a material devoid of cellular components while retaining a collagenous matrix and epitopes for cell binding. Materials for further recellularization and implantation should not contain any toxic residues of decellularization agents or immunogenic epitopes. The definition of acellularity in the context of whole liver decellularization needs further refinement. Firstly, the criteria proposed by Crapo, based on DNA quantification, do not appear to be sufficient for the safe implementation of the model as they do not include potentially toxic or immunogenic substances that may remain in the scaffold after decellularization [23]. Secondly, given the size and perfusion dynamics of porcine and human livers, the definition should include matrix assessment at different anatomical sites. However, sampling of the liver during the process can cause significant damage to the matrix, that can impede decellularization by causing uncontrolled fluid leakage. Therefore, the development of non-destructive techniques for organ evaluation and their validation with destructive techniques is of critical importance.

Immersion and agitation techniques are not effective when considering full-size parenchymal organs, and their effectiveness is limited to small, flat tissues with few cells. The most commonly applied perfusion technique, using a peristaltic pump, does not appear to be optimal for whole organ decellularization, as approximately one third of its working cycle counteracts onward perfusion. As shown in the Supplementary Materials (Table S1) the peristaltic pump causes oscillating movement of the perfusion fluid within the organ, which may prolong matrix exposure to detergents. Furthermore, most of the protocols report a wide range of flow rates that are rarely optimized to the weight of the organ. Gravity perfusion systems provide a controlled and reproducible environment for the decellularization of parenchymal organs. Such a technique, coupled with an automated pressure system, offers an optimized protocol that minimizes detergent exposure time. Given the phenomenon of outlet obstruction with cellular debris, the system could theoretically adjust the level of fluid in order to maintain stable parenchymal pressure during perfusion. Such a system that allows for interstitial fluid measurements in the liver decellularization model has been described by Moran et al. [98]. However, their system was not reciprocally coupled with perfusion parameters. This model requires further studies to assess its effectiveness in removing cellular debris. It may also be coupled with the concept of decellularization cycles to provide comparability. Interstitial pressure control during perfusion may also be particularly important during the recellularization phase. In addition, matrix assessment at defined time points could allow for the application of time-to-event analysis to better understand and further optimize decellularization protocols.

In the context of liver tissue decellularization, the maintenance of an alkaline pH of the perfusion fluid and the perfusion step with DNase seem to be particularly valid due to the polyploidy of the hepatocytes. DNA denaturation and disintegration can help to achieve acellular status in the DNA phase of the decellularization cycles and further minimize the time required to obtain a decellularized matrix. Conversely, the concept of decellularization cycles can be applied in reverse during the recellularization phase, when the biofabricated organ is evaluated microscopically at defined time intervals. Given the complexity of cell removal and restoration, such a standardized approach would lead to a better understanding of the processes and allow comparisons to be made. In addition, the need for frequent matrix assessment and translation into human organs means that, in our opinion, rodent models do not appear to be sufficient for further studies in this area. The similarity of the porcine liver to the human organ and the feasibility of apoptosis induction only in the animal model provide a good rationale for choosing the pig liver as a standard for further studies.

A thorough understanding of the advantages and disadvantages of decellularization agents can help in the designing of a protocol that minimizes matrix damage and improves its biocompatibility. The concept of apoptosis induction may help to reduce the decellularization time while protecting the matrix from damage from both intracellular enzymes and detergent exposure.

## 5. Conclusions

Existing methods for producing acellular matrices are based on whole organ perfusion with decellularization agents, most of which contain detergents. Both uncontrolled perfusion and the aggressive nature of the detergents used may be responsible for excessive matrix damage, affecting its biocompatibility and immunogenicity and thus limiting its potential clinical implementation. In addition, the lack of a widely accepted definition of acellularity limits the comparability of different protocols. Herein, we presented a novel concept of decellularization cycles that may allow for a better understanding of the decellularization dynamics and increase comparability. As an alternative to standard peristaltic pump perfusion, we suggest that pressure-driven protocols that have been shown to be more effective in animal models should be favored as they provide a steady and gentle flow through the organ. The development of non-invasive means of assessing decellularization could allow frequent matrix evaluation during decellularization cycles to better understand the process without damaging the organ. Apoptosis-induced decellularization constitutes a promising improvement in the field as it minimizes the need to expose the matrix to decellularization agents, with minimal ECM damage mediated by the released intracellular enzymes and vessel clogging by DNA strands remaining in the apoptotic bodies.

## Data Availability

No new data were created or analyzed in this study.

## Supplementary Materials

The following supporting information can be downloaded at https://www.mdpi.com/article/10.3390/biom15010098/s1, Table S1: Summary of the decellularization protocols; Video S1: Slow motion video of the peristaltic pump showing significant backflow during the pumping cycle [Author’s own work].
