# Supplementary material for: Decellularized Liver Matrices for Expanding the Donor Pool—An Evaluation of Existing Protocols and Future Trends
Source: Biomolecules. 2025 Jan 10;15(1):98. doi: 10.3390/biom15010098 (PMC11762870; doi:10.3390/biom15010098)
Supplement: Supplementary file 1 [file biomolecules-15-00098-s001.zip › Supplementary Material Table S1.pdf]

Table S1 Summary of decellularization protocols in animal and human models.

Species	Total time	Freezing at – 80℃	Perfusion route	Flow rate	Wash solution 1	Decellularization agent 1	Wash solution 2	Decellularization agent 2	Wash solution 3	Decellularization agent 3	Wash solution 4	Reference
Mouse	200 min	No	PV	3.8 ml/min	PBS<br>(10 min)	1% Triton X-100<br>(90 min)	-	1% SDS<br>(90 min)	PBS<br>(10 min)	-	-	Diedrich<br>(2024)
Rat	21 – 33 h	Yes	PV	5 ml/min	PBS<br>(1 h)	0.05% EGTA +<br>0.05% trypsin<br>(2 h)	-	0.05% EGTA +<br>0.5 % Triton X-100<br>(12 - 24 h)	PBS<br>(6 h)	-	-	Hirukawa<br>(2024)
Rat	5 h	No	PV & IVC	7 ml/min	PBS +<br>doxycycline<br>(1 h)	0.1% SDS<br>(3 h)	PBS<br>(1 h)	-	-	-	-	Kasravi<br>(2024)
Rat	585 min	No	PV<br>or HV<br>or BD	1 ml/min	KHB<br>(1 h; flow =<br>0.5 ml/min)<br><br>dH2O<br>(30 min)	1% SDS<br>(1 h);<br>0.5% SDS<br>(45 min);<br>0.1% SDS<br>(30 min)	-	0.5% Triton X-100<br>(30 min);<br>0.2% Triton X-100<br>(45 min);<br>0.1% Triton X-100<br>(1 h);<br>0.1% SDC<br>(30 min)	dH2O<br>(1 h)	DNAse<br>(15 min);<br>dH2O<br>(1 h; flow =<br>1.5 ml/min);<br>PBS<br>(1 h; flow =<br>1.5 ml/min)	-	Nooshin<br>(2023)
Rat	750 min	Yes	PV	Gravity<br>perfusion<br>system<br>(7 ml/min)	-	1% Triton X-100;<br>2% Triton X-100;<br>3%Triton X-100;<br>0.1% SDS<br>(3 h each)	PBS<br>(30 min)	-	-	-	-	Morales-<br>Guerrero<br>(2022)
Rat	1010 min	Yes	PV		dH2O<br>(4 x15 min;<br>5 ml/min,<br>10 ml/min,<br>15 ml/min,<br>20 ml/min)	1% Triton X-100 +<br>0.1% NH_4OH<br>(1 h, 15 ml/min;<br>2 h, 20 ml/min)	dH2O<br>(40 min,<br>5 ml/min;<br>15min,<br>15 ml/min;<br>45 min,<br>20 ml/min)	0.1% PAA in 4%<br>alcohol<br>(40 min,<br>5 ml/min)<br>0.1% PAA<br>(30 min)	dH2O<br>(8 h,<br>5 ml/min)	-	PBS + 1%<br>penicillin/<br>streptomycin<br>(2 h,<br>6 ml/min)	Meng<br>(2019)

Species	Total time	Freezing at – 80℃	Perfusion route	Flow rate	Wash solution 1	Decellularization agent 1	Wash solution 2	Decellularization agent 2	Wash solution 3	Decellularization agent 3	Wash solution 4	Reference
Rat	960 min	Yes	PV	6 ml/min	PBS (1 h)	0.05% trypsin + 0.05% EGTA (2 h)	-	0.5% Triton X-100 + 0.05% EGTA (from 3 to 12 h)	PBS (1 h)	-	-	Watanabe (2019)
Rat	1635 min	Yes	PV	1.2 ml/min	PBS (8 h)	0.01% SDS (5, 10, 15 min) + PBS (3 x 1 h) 0.01% SDS (8 h), 0.1% SDS (4 h), 0.2% SDS (1 h), 0.5% SDS (1 h)	dH2O (15 min)	1% Triton X-100 (30 min)	PBS (1 h)	-	-	Devalliere (2018)
Rat	1620 min	No	PV	1 ml/min	PBS (wash)	0.02% trypsin + 0.05% EGTA (1 h)	-	1% Triton X-100 + 0.05% EGTA (24 h)	-	0.1% PAA (2 h)	PBS (wash)	Kojima (2017)
Rat	2715 min	Yes	PV	1.2 ml/min	PBS (8 h)	0.01% SDS (5, 10, 15 min) + PBS (3 x 1 h) 0.01% SDS (8 h), 0.1% SDS (4 h), 0.2% SDS (1 h), 0.5% SDS (1 h)	dH2O (15 min)	1% Triton X-100 (30 min)	PBS (1 h)	-	-	Geerts (2016)
Rat	200 min	No	PV or HA	5 ml/min + oscillating pressure conditions (respirator with 15 bpm/ 26 mbar)	PBS (10 min)	1% Triton X-100 (90 min)	-	1% SDS (90 min)	PBS (10 min)	-	-	Hillebrandt (2015)
Rat	4410 min	Yes	PV	1 ml/min	PBS (8 h)	2 x 0.1% SDS (24 h) and 0.2% SDS (12 h); 0.1% Triton X-100 (30 min)	PBS (2 h)	PBS + 0.1% PAA + 4% EtOH (3 h)	PBS (rinse)	-	PBS + 250 μg/mL amphotericin B, 200 U/mL penicillin and 200 mg/mL streptomycin (7 d)	Bruinsma (2015)

Species	Total time	Freezing at – 80℃	Perfusion route	Flow rate	Wash solution 1	Decellularization agent 1	Wash solution 2	Decellularization agent 2	Wash solution 3	Decellularization agent 3	Wash solution 4	Reference
Rat	260 min	No	PV or IVC	10 ml/min	D-Hanks medium (30 min)	1% Triton X-100 (30 min)	-	1 cycle of 1% SDS, 0.5% SDS, and 0.1% SDS (50 min)	PBS (wash)	-		Pan (2014)
Rat	210 min	No	PV	5 ml/min	PBS (100 ml)	1% SDS or 1% Triton X-100 + 0.05% sodium hydroxide (2 h)	dH2O (30 min; 6.67 ml/min), PBS (1 h)	-	-	-	-	Ren (2013)
Mouse	1800 min	Yes	IVC	8 ml/min	PBS (until thawed)	0.02% trypsin + 0.05% EGTA (2 h)	dH2O (15 min), 2 x PBS (15 min)	3% Triton X-100 + 0.05% EGTA (24h)	dH2O (15 min), 2 x PBS (15 min), dH2O (30 min) 2 x PBS (30 min)	0.1% PAA + 4% EtOH (1 h)	2 x PBS (15 min) dH2O (15 min)	Soto-Gutierrez (2011)
Rat	8745 min	Yes	PV	1 ml/min	PBS (72 h)	Alternating cycles of 0.01% SDS, 0.1% SDS, 1% SDS (24 h) and dH2O (15 min)	-	1% Triton X-100 (30 min)	PBS (1 h)	-	-	Uygun (2010)
Rat	322 min	No	IVC	5 ml/min	PBS (20 min)	PBS + 1% Triton X-100, 2% Triton X-100, 3% Triton X-100 (1 h)	-	PBS + 0.1% SDS (1 h)	PBS (300 ml), then FBS (2 min)	-	All perfusion solutions contained 1% antibiotic/ antimycotic	Shupe (2010)
Porcine	4200 min	Yes	PV	50 ml/min	dH2O (1 h), 2 x PBS (1 h)	0.2% trypsin + 0.5% EDTA + 0.05% NaN3 (12 h)	-	3% Triton X-100 + 0.05% EGTA + 0.05% NaN3 (24 h)	-	4% SD (34 h)	-	Alaby Pinheiro Faccioli (2022)

Species	Total time	Freezing at – 80℃	Perfusion route	Flow rate	Wash solution 1	Decellularization agent 1	Wash solution 2	Decellularization agent 2	Wash solution 3	Decellularization agent 3	Wash solution 4	Reference
Porcine	720 – 840 min	Yes	PV and HA	Pressure-controlled perfusion (max. 120 mmHg for HA)	dH2O (50 l)	5 x 4% Triton X-100 + 1% NaN3 (120 min with 10 l solution) or 2 x 4% Triton X-100 (120 min), then 1% SDS (120 min)	dH2O (50 l); storage in dH2O (10 - 14 d)	DNAse at 37℃ (120 min)	-	-	-	Willemse (2020)
Porcine	n/d	Yes (at -20℃)	PV	n/d	dH2O	1% SDS followed by 1% Triton X-100	-	-	-	Crosslinking	PBS + 10% v/v penicillin/ streptomycin	Gao (2019)
Porcine	n/d	No	PV	Pressure-perfusion (12 mmHg)	0.6% SDS (48 h)	dH2O (wash)	0.1% PAA (n/d)	PBS (wash)	-	-	-	Mao (2017)
Porcine	420 min	Yes	PV and HA	Pressure-controlled (PV: 60 mmHg; HA: 120 mmHg)	dH2O (30 min)	2 x 1% Triton X-100 (90 min)	-	2 x 1% SDS (90 min)	dH2O (30 min)	-	-	Struecker (2015)
Porcine	1680 min	Yes (at -20℃)	PV	200 ml/min	dH2O (1 h)	1% SDS or 1% Triton X-100 or 1% PAA or 1% NaDOC (24 h)	PBS (3 h)	-	-	-	-	Wang (2015)
Porcine	1320 min	Yes	PV	Pressure-controlled (20 mmHg)	PBS (1 h)	1% SDS (10 l)	PBS (rinse)	DNAse (2 h)	PBS (rinse)	-	-	Bühler (2015)
Human	32 h	Yes (at -20℃)	PV and HA	Pressure-controlled (max. 120 mmHg for HA)	dH2O (50 l)	4% Triton X-100 (120 min)	-	10 x 4% Triton X-100 (120 min)	dH2O (100 l); storage in dH2O (10 - 14 d)	DNAse at 37℃ (8 h)	-	Willemse (2020)

Species	Total time	Freezing at – 80℃	Perfusion route	Flow rate	Wash solution 1	Decellularization agent 1	Wash solution 2	Decellularization agent 2	Wash solution 3	Decellularization agent 3	Wash solution 4	Reference
Human	4 – 6 days	No	PV and HA	60 ml/min; 350 ml/min (at day 3)		4% Triton X-100 + 1% NH4OH	0.9% NaCl (1 l, 30 ml/min)	DNAse-I 2 mg/l (1 l, 8 h)	0.9% NaCl (1 l, 30 ml/min)	-	-	Verstegen (2017)
Human	2 – 6 weeks	Yes	PV	0.2 - 0.3 ml/min/g	-	0.025% trypsin - EDTA (1 d); 0.01 - 1% SDS (1 d); 0.025% trypsin-EDTA (1 d); 3% Triton X-100 (4 d); 0.025% trypsin-EDTA (1 d); 1% SDS (5 d); 0.1% PAA/PBS (1 d)	-	-	-	-	-	Mazza (2015)

Abbreviations: BD - bile duct; dH2O - distilled water; EDTA - ethylenediaminetetraacetic acid; EGTA - ethylene glycol tetraacetic acid; EtOH - ethanol; FBS - fetal bovine serum; HA - hepatic artery; IVC - inferior vena cava; KHB - Krebs-Henseleit buffer; NaDOC - sodium deoxycholate; n/d – no data; PAA - peracetic acid; PBS - phosphate-buffered saline; PV - portal vein; SD - sodium deoxycholate; SDC - sodium deoxycholate; SDS - sodium dodecyl sulfate.
